# IgG4-Related Disease: A Rare Case of Simultaneous Lung and Retroperitoneal Involvement

**DOI:** 10.7759/cureus.28521

**Published:** 2022-08-29

**Authors:** Filipa Duarte, Andreia Tavares, Daniela Soares, José Meireles

**Affiliations:** 1 Internal Medicine, Centro Hospitalar de Entre o Douro e Vouga, Santa Maria da Feira, PRT

**Keywords:** corticosteroids, immunosuppressive therapy, pulmonary consolidation, retroperitoneal fibrosis, igg4, immunoglobulin g4-related disease

## Abstract

Immunoglobin G4-related disease is a progressive immune-mediated fibroinflammatory condition that can affect any organ, causing a tumor-like swelling appearance.

We present a case of a 57-year-old male who presented with a one-month history of weight loss, constant abdominal pain with dorsal irradiation, night sweats, and respiratory symptoms. CT scan revealed multiple mediastinal and retroperitoneal adenopathies, right pulmonary consolidation, and retroperitoneal fibrosis. Transthoracic pulmonary biopsy and excisional cervical lymph node biopsy revealed fibroinflammatory disease related to IgG4, with normal serum IgG4. The patient presented a good response to glucocorticoids, a clinical characteristic of this disease.

The diagnosis of immunoglobin G4-related disease is challenging, due to the nonspecific clinical manifestations, requiring a high level of suspicion in order to perform the appropriate immunohistochemical examination.

## Introduction

Immunoglobin G4-related disease (IgG4-RD) is an insidiously progressive immune-mediated fibroinflammatory condition [1]. Its exact prevalence is unknown since the recognition of the disease continues to grow with many undiagnosed cases. However, there seems to be a male predominance and a peak between the fifth and seventh decade. The pancreas, major salivary glands, lacrimal glands, retroperitoneum, and lymphatic ducts are the most frequently affected organs, which often present a tumor-like swelling appearance generally detected by imaging studies [2]. The affected tissues present dense lymphoplasmacytic infiltrations with a predominance of IgG4-positive plasma cells, storiform fibrosis, obliterative phlebitis, and modest tissue eosinophilia [3]. The clinical manifestations are nonspecific, such as lymphadenopathy and weight loss, and the diagnosis is based upon the combination of characteristic histopathologic, clinical, serologic, and radiologic findings.

## Case presentation

A 57-year-old male with a previous history of essential hypertension, type 2 diabetes mellitus, dyslipidemia, smoking, and regular alcohol consumption (about 50 grams per day) presented a one-month history of weight loss (approximately 10 kilograms, more than 10% body weight), constant abdominal pain radiating dorsally and night sweats. He also reported marked asthenia and anorexia, pleuritic pain as well as dyspnea for minor exertion. He denied blood loss and gastrointestinal and genitourinary symptoms. Physical examination showed a globose, soft abdomen with normal bowel sounds and no palpable masses, but with diffuse pain on palpation and tenderness in the epigastric and left lumbar regions. There were no palpable adenomegalies. Lactate dehydrogenase, erythrocyte sedimentation rate, C-reactive protein, and total serum proteins were increased (table 1).

**Table 1:** Analytic results

Parameter	Value	Reference value
Erythrocyte sedimentation rate	88 mm	< 15
Lactate dehydrogenase	698 U/L	45 – 90
C-reactive protein	82 mg/L	< 5,0
Total serum proteins	9,1 g/dL	6,4 – 8,3
Beta-2microglobulin	7,31 mg/L	< 2,64
Angiotensin-converting enzyme	78.1 U/I	13,3 – 63,9
C3	68 mg/dL	82 – 185
C4	3 mg/dL	15 – 53
C1q	24,0 mg/dL	10,0 – 25,0
IgA	408 mg/dL	63 – 484
IgG	3649 mg/dL	540 – 1882
IgM	571 mg/dL	22 - 240
IgG4	35,0 mg/dL	9,0 – 104,0

**Figure 1:**
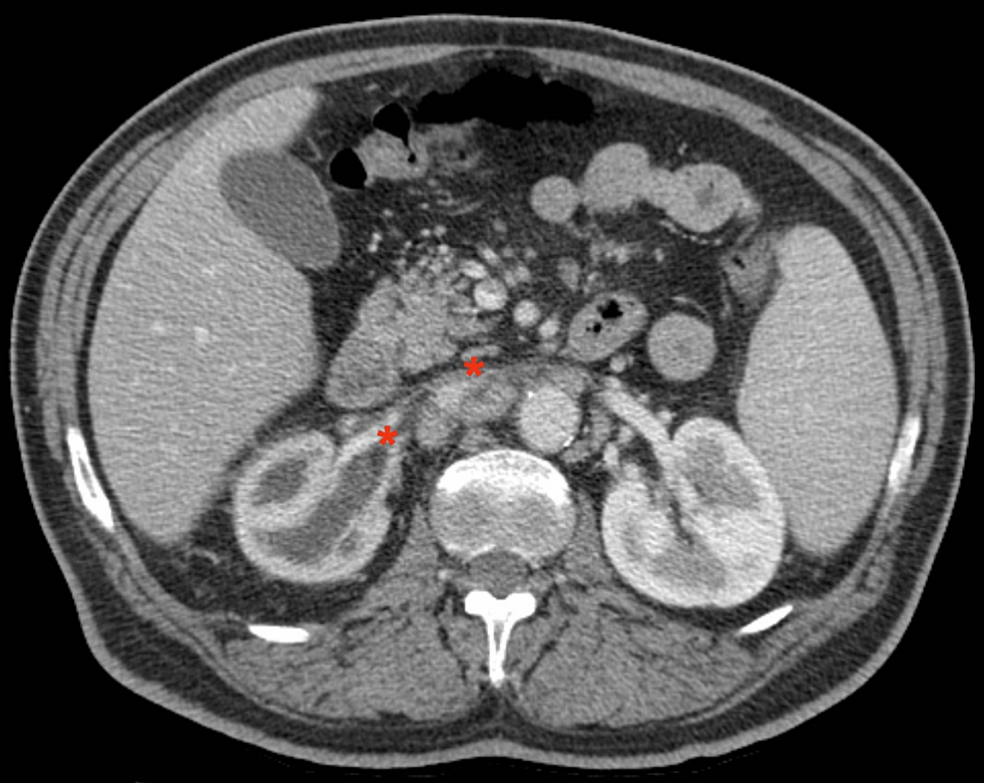
Abdominal CT-scan revealing right renal atrophy with nonspecific ureteral and pyelocaliceal ectasia and retroperitoneal fibrosis involving the right ureter

Computed tomography (CT) scan of the chest and abdomen revealed multiple mediastinal and retroperitoneal adenopathies, and ground-glass opacities in the right upper lobe with associated interlobular and peribronchovascular interstitial thickening, probably related to interstitial pneumopathy. It also showed right renal atrophy with nonspecific ureteral and pyelocaliceal ectasia caused by an obstructive process associated with some degree of retroperitoneal fibrosis (Figure 1). He was admitted to the Internal Medicine ward and completed seven days of antibiotic therapy due to pneumonia, with a favorable initial response. Extensive diagnostic workup (during hospitalization and after discharge) excluded human immunodeficiency virus, hepatitis B and hepatitis C virus infections, as well as tuberculosis (active and latent) and toxoplasmosis revealed hypocomplementemia of C3 and C4, high beta-2microglobulin, angiotensin-converting enzyme, IgG and IgM, with normal IgG4; normal serum protein electrophoresis and negative autoimmune study (rheumatoid factor, antinuclear antibodies, antineutrophil cytoplasmic autoantibodies, anti-double-stranded DNA antibodies, anti-glomerular basement membrane). Positron emission tomography showed multiple hypermetabolic adenopathies above and under the diaphragm, as well as areas of parenchymal densification scattered in the right upper lobe and middle lobe of the lungs. Peripheral ganglion needle biopsy excluded cancer and lymph node tuberculosis and immunophenotyping excluded non-Hodgkin’s lymphoma. 

**Figure 2:**
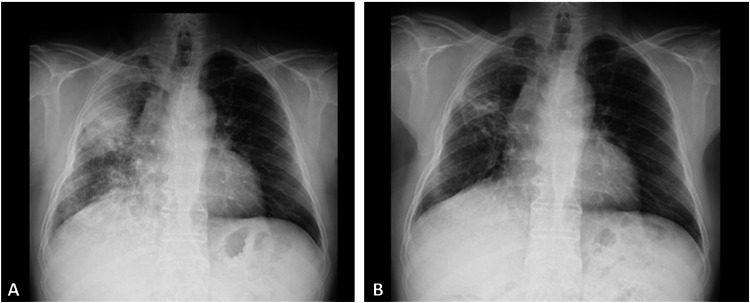
Evolution of the infiltrate in the middle lobe of the right lung on chest radiography (posteroanterior view) A - before treatment; B - after 4 weeks of corticosteroid therapy.

Three months after discharge, the patient presented worsening of the right lung consolidation and progression of retroperitoneal fibrosis, with minimal symptoms detected in routine follow-up imaging. He was admitted for a transthoracic pulmonary biopsy and excisional cervical lymph node biopsy which revealed fibroinflammatory disease related to IgG4. The patient started high-dose glucocorticoids (prednisolone 1mg/kg) with good response, with partial resolution of lung densification (Figure 2) and reduction in the size of adenopathies. There was no disease progression during glucocorticoid tapering. 

The patient had uncontrolled diabetes mellitus due to glucocorticoid therapy. Despite several insulin therapy adjustments and corticoid tapering (at that time, 0,5mg/kg, 30mg per day), the patient presented diabetic ketoacidosis and was admitted to the intensive care unit where he developed septic shock due to severe *Strongyloides stecoralis* infection and *Escherichia coli* bacteriemia. He did not respond to the treatment, presented refractory shock, and died.

## Discussion

IgG4-RD is a systemic disorder that is often confused with cancer, due to its nonspecific symptoms and the diffuse enlargement of the affected organs. There is growing evidence that its pathogenesis has an autoimmune basis and multiple organs are affected in 60-90 percent of cases [4]. Our patient presented retroperitoneal fibrosis, one of the most common manifestations, as well as lymph node and pulmonary involvement, a rare manifestation. High levels of serum IgG4 are found in 60-70 percent of patients [5]. However, a small portion presents a normal serum concentration of IgG4 [6, 7] (which is not an exclusion criterion), and therefore biopsy remains the cornerstone of the diagnosis. A core needle biopsy is often adequate, but fine-needle aspirates do not provide adequate tissue [8, 9]. A high clinical suspicion is essential in order to perform the appropriate immunohistochemical examination. The initial therapy is based on glucocorticoids and its responsiveness to treatment is another clinical characteristic of IgG4-RD [10]. Most patients require an additional immunosuppressive, sometimes including a biological therapy such as rituximab, in order to achieve disease remission [11], and to minimize the adverse effects of therapy with glucocorticoids. Furthermore, many patients present disease flares during or after glucocorticoid tapering.

## Conclusions

IgG4-RD is a fibroinflammatory disorder that can affect any organ, causing a lymphoplasmacytic infiltrate rich in IgG4-positive plasma cells, which leads to a variable degree of fibrosis. The diagnosis depends on a combination of characteristic histopathologic, clinical, serologic, and radiologic findings. Glucocorticoids are the first line of therapy and the responsiveness to treatment is another clinical characteristic of the disease. Many cases require an additional immunosuppressive, sometimes including a biological therapy, to achieve disease remission and minimize the adverse effects of glucocorticoids.

The nonspecificity and multiplicity of symptoms make its diagnosis challenging and, therefore, is important to raise awareness of the disease.
